# Characteristic Photoprotective Molecules from the Sphagnum World: A Solution-Phase Ultrafast Study of Sphagnic Acid

**DOI:** 10.3390/molecules28166153

**Published:** 2023-08-21

**Authors:** Michael Hymas, Irene Casademont-Reig, Stéphane Poigny, Vasilios G. Stavros

**Affiliations:** 1 Department of Chemistry, University of Warwick, Coventry CV4 7AL, UK; michael.hymas@warwick.ac.uk (M.H.); irene.casademont.reig@vub.be (I.C.-R.); 2 Department of General Chemistry (ALGC), Vrije Universiteit Brussel (VUB), Pleinlaan 2, 1050 Brussels, Belgium; 3 Mibelle Group Biochemistry, Mibelle AG, Bolimattstrasse 1, CH-5033 Buchs, Switzerland; 4 School of Chemistry, University of Birmingham, Edgbaston, Birmingham B15 2TT, UK

**Keywords:** sphagnum moss, avobenzone, sunscreen, photoprotection, ultrafast spectroscopy, photophysics, photochemistry

## Abstract

A natural UV-absorbing chromophore extracted from sphagnum mosses, sphagnic acid, is proposed as a new natural support to chemical UV filters for use in cosmetic applications. Sphagnic acid is structurally related to the cinnamate family of molecules, known for their strong UV absorption, efficient non-radiative decay, and antioxidant properties. In this study, transient electronic absorption spectroscopy is used, in conjunction with steady-state techniques, to model the photodynamics following photoexcitation of sphagnic acid in different solvent systems. Sphagnic acid was found in each system to relax with lifetimes of ~200 fs and ~1.5 ps before generating a *cis*-isomer photoproduct. This study helps to elucidate the photoprotective mechanism of a new potential natural support to sunscreens, from a unique plant source.

## 1. Introduction

Solar ultraviolet radiation (UVR), particularly that in the UV-B region (280–315 nm), is well known to cause damage to the skin [1,2]. Specific health risks arising from overexposure to UV-B include malignant melanoma and squamous cell carcinoma [3]_._ In a continuing effort to combat these adverse health effects, synthetic sunscreens have been developed with the requisite absorption features to protect against UVR not already absorbed by atmospheric ozone [4].

Sunscreens are characterised as either inorganic or organic filters: inorganic filters achieve photoprotection by scattering and absorbing incident radiation, whereas organic filters absorb and ideally dissipate the energy through non-radiative decay [5,6]. Controversies surrounding existing sunscreen formulation components, especially certain UV filters shown to generate harmful photoproducts, have stimulated research into novel organic molecules capable of photoprotection [7,8]. To facilitate effective sunscreen molecule design, not only are new strongly absorbing species being designed and synthesised, but mechanisms of action of existing sunscreen molecules have been investigated and relaxation schemes of potential UV filter compounds following UVR absorption are being analysed [7,9,10,11,12].

**Figure 1:**
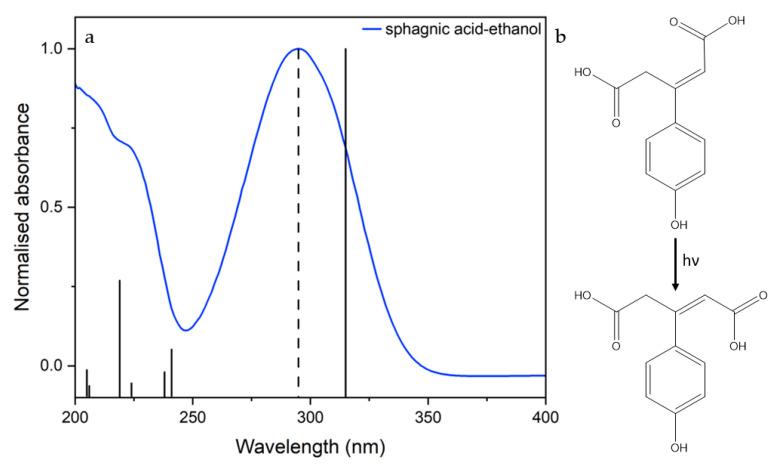
(**a**) UV-vis spectrum of sphagnic acid in ethanol (blue); dotted line shows pump wavelength selected for transient electronic absorption spectroscopy (TEAS) (295 nm); calculated vertical excitations for sphagnic acid in ethanol (solid black). (**b**) Schematic of *trans*-*cis* photoisomerisation of sphagnic acid.

Sphagnic acid (or *trans*-sphagnum acid, (*E*)-3-(4-hydroxyphenyl)pent-2-enedioic acid), hereafter abbreviated to SphA, has recently been proposed for support to artificial UV filters [13]. Sphagnol, a substance initially extracted from the cell walls of sphagnum mosses in the late 19th century, contains a mixture of phenols (including SphA) and was used in the treatment of skin diseases and in wound dressings due to its antiseptic and antifungal properties [14]. SphA was first identified in the 1970s and characterised by analysis of the cell walls of *Sphagnum magellanicum* but has since been found in other sphagnum mosses [15,16]. This molecule is believed to impart UVR photoprotection to sphagnum mosses, absorbing harmful UV-B radiation [13,16,17]. SphA shares many structural characteristics with other plant UV filters, such as the phenylpropanoid moiety present in sinapic acid and its derivatives [13,18,19]. Sinapic acid and its derivatives also exhibit strong UV absorption and antioxidant properties [13,20]. As such, SphA has been suggested for use in cosmetic formulations as part of a sphagnum moss extract that could be obtained either by classical extraction or produced by biotechnological methods [13,21]. The analysis of this compound provides a new avenue for the investigation of natural UV filters. In contrast to current plant-inspired UV filters derived from organisms that grow in direct sunlight, SphA is extracted from mosses which typically thrive in damp and dark environments [15,16,22]. In addition to this, SphA’s absorption profile (see Figure 1) means it absorbs in approximately the same region as the *diketo* tautomer form of the widely used UV filter avobenzone [23]. The *diketo* form of avobenzone, formed from the photo-induced tautomerisation of the *enol*, is well known to be unstable, generating harmful photoproduct radicals via a Norrish Type I cleavage when exposed to UVR [24,25,26,27]. The use of SphA or a biotechnologically recovered sphagnum extract in blends containing avobenzone could prevent the generation of these harmful photoproducts by absorbing some of the incident photon flux that would otherwise initiate the cleavage reaction, thereby providing nature-based support to an extensively used chemical UV filter [23].

SphA, being structurally related to existing UV filters whose photoprotection mechanisms have already been studied in the gas and solution phase, is likely to follow the same proposed relaxation mechanism [28,29]. This non-radiative decay (NRD) mechanism can be summarised thus: 1^1^ππ* → S_0_ (v > 0) → S_0_ (v = 0) (*trans* or *cis*). Following photoexcitation to the first excited singlet ππ* state, the population transfers back to the S_0_ singlet ground state mediated by rapid motion out of the Franck–Condon region to a 1^1^ππ*/S_0_ conical intersection (CI), at which photoisomerisation occurs to form either the *cis* isomer or the starting *trans* isomer, before vibrationally cooling down the S_0_ potential energy surface [28,30]. According to this identified mechanism, the only photoproduct generated should be the *cis* isomer of the molecule (see Figure 1) [28].

In the present study, we seek to confirm this NRD mechanism proposed following photoexcitation at the absorbance peak maximum (UV-B, 1^1^ππ*) in three solvent systems: ethanol, acetonitrile and dioxane (in order of decreasing polarity). We also seek to extract lifetimes attributed to each step of the ultrafast relaxation process. To do this, the photodynamics were probed using femtosecond transient electronic absorption spectroscopy (fs-TEAS) and analysed via the sequential global fitting software Glotaran [31]. The photostability of SphA was also investigated using steady-state spectroscopies.

## 2. Results

### 2.1. Transient Electronic Absorption Spectroscopy (TEAS)

**Figure 2:**
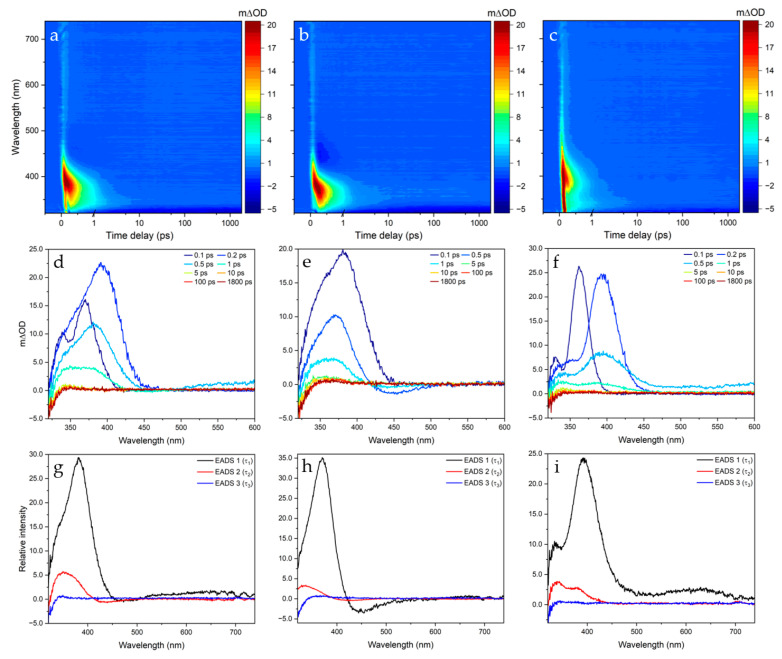
Two-dimension heat maps of the TAS (**a**–**c**), TAS at selected pump-probe time delays (**d**–**f**) and EADS extracted from Glotaran fitting (**g**–**i**). SphA in ethanol was photoexcited at 295 nm (**a**,**d**); SphA in acetonitrile was photoexcited at 294 nm (**b**,**e**); SphA in dioxane was photoexcited at 294 nm (**c**,**f**). The colour maps and selected TAS depict the change in optical density (OD).

The solution-phase photodynamics of SphA in each of the three solvent systems (ethanol, acetonitrile and dioxane) was monitored via TEAS. The (pump) photoexcitation wavelength (assigned as S_1_ ← S_0_ by TDDFT calculations) was centred at λ_max_ for the first peak in the UV-visible absorption spectrum of SphA in each solvent system (see Supplementary Figures S1–S3). Figure 2 shows the collected transient absorption spectrum/spectra (TAS) for each system studied, with SphA-ethanol photoexcited at 295 nm and SphA-dioxane and SphA-acetonitrile photoexcited at 294 nm. The TAS collected for each system, presented in the form of two-dimensional heat maps (Figure 2a–c), TAS at selected pump-probe time delays (Figure 2d–f) and evolution-associated difference spectra (EADS) extracted from fitting (Figure 2g–i), all show similar features and associated lifetimes. These are, to a greater or lesser extent, features typically observed in TAS: excited state absorption (ESA) in which the initially populated excited state is further excited by the white light probe, ground state bleach (GSB) in which the ground state population is depleted by the pump pulse and stimulated emission (SE) in which the initially populated excited state is stimulated to relax back to the electronic ground state by the white light probe. Further details pertaining to these features can be found in the work by Berera et al. [32]. At first sight, the TAS collected show that GSB recovery is achieved in a few ps; the ESA (centred at ~380 nm in all systems) and weak SE (centred at ~450 nm in acetonitrile) almost completely diminish within ~2 ps for each system. To obtain quantitative insight into the number of processes occurring and their respective lifetimes, a sequential global fitting technique was employed using the software package Glotaran. The accuracy of the fits generated is reflected in the residuals produced for each two-dimensional heat map of the TAS (see Supplementary Figures S4–S6). The results of these fitting procedures are summarised in Table 1. Three lifetimes were identified in each case, the third of which accounts for the incomplete GSB recovery reflecting the photoproduct absorption profile; see below for further discussion. Instrument response was extracted experimentally by measurement of solvent-only TAS under identical pump-probe conditions to elicit the solvent response to photoexcitation (see Supplementary Figures S7–S9).

### 2.2. Steady-State Spectroscopy

**Figure 3:**
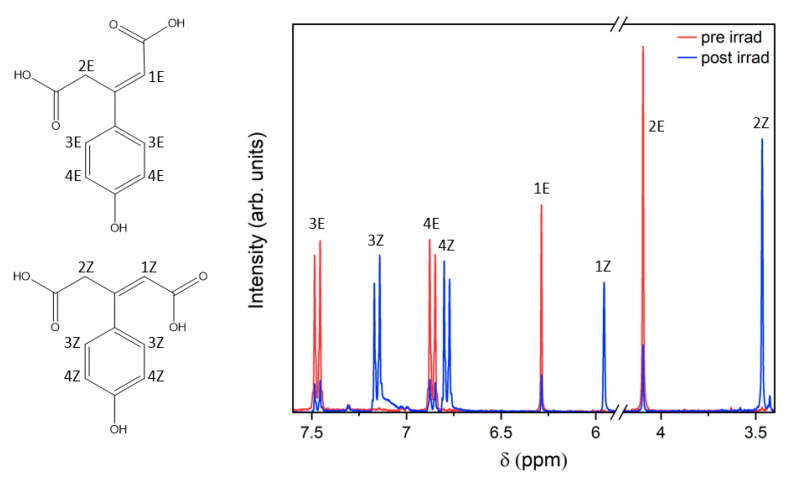
^1^H NMR spectra showing SphA in d_3_-acetonitrile pre- (red) and post-irradiation (blue) for 3 h with a solar simulator. Signals in the spectral window shown are labelled with their respective assignments on the *trans* and *cis* isomer structures of SphA (see Supplementary Figures S16–S19 for full spectra).

^1^H and COSY NMR spectra were obtained pre- and post-3 h irradiation of SphA in deuterated acetonitrile with a solar simulator (see Supplementary Figures S10–S13). These spectra were taken to check for photoproducts potentially resolvable by ^1^H shift or by J-coupling values. The key features of the ^1^H NMR spectra are shown in Figure 3.

**Figure 4:**
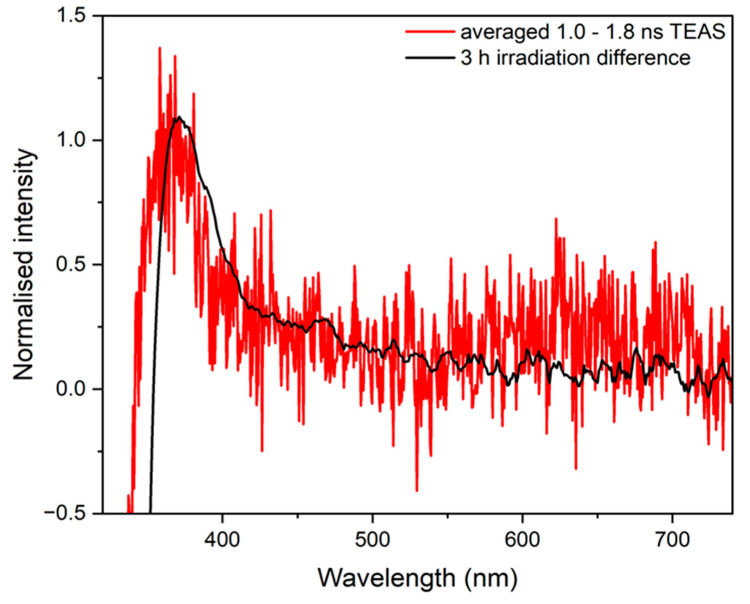
Normalised UV-visible difference spectra following irradiation of sphagnic acid in acetonitrile with a solar simulator for 3 h (black) overlaid with normalised average Δ*t* = 1.0–1.8 ns TAS (red).

TAS collected at late pump-probe time delays of our experiment (Δ*t* = 1.0–1.8 ns) and UV-visible difference spectra of SphA in acetonitrile collected after 3 h irradiation at 294 nm are displayed together in Figure 4. These data are compared to check that photoproducts generated on the ps timescale correspond to long-lived photoproducts formed from steady-state irradiation. To add, UV-visible spectra were collected at various time delays of irradiation with a solar simulator, spanning 3 h, to determine the photostability of SphA in each solvent (see Supplementary Figures S14–S16).

## 3. Discussion

From the two-dimensional heat maps in Figure 2, a positive signal attributed to ESA is present in all three solvent systems studied, centred at 390 nm in ethanol, at 380 nm in acetonitrile and at 400 nm in dioxane. Within a few hundred femtoseconds the ESA blue shifts into a new ESA feature. This blue shift indicates that the absorbing molecule is undergoing geometry relaxation in the excited state, as described in previous models [33]. The ESA decays to baseline within ~2 ps in each case. A slight negative feature centred at 450 nm is also visible in the acetonitrile TAS, which decays with the same lifetime as the ESA, suggesting this may reflect SE from an excited state.

TAS at much longer Δ*t* possess a slight long-lived positive absorption feature, evident in the selected time traces from 5 ps to 1.8 ns; the averaged TAS from Δ*t* = 1.0–1.8 ns is shown in Figure 4 (red trace) as an exemplar. Excited state photodynamics were considered to have finished by 1.0 ns, so any signal afterwards arises purely from a long-lived photoproduct. If the mechanism of relaxation follows the same pathway as that identified in previous studies (i.e., *trans* to *cis* photoisomerisation) then this positive feature would be assigned to the formation of the stable *cis* isomer, which is formed on the ps timescale [28,34,35]. A difference UV-visible spectrum of the system pre- and post-irradiation with a solar simulator (Figure 4, black trace) shows the difference absorption profile between the stable photoproduct formed and starting material. This is the steady-state analogue of the long-time delay (i.e., 1.0–1.8 ns) TAS obtained under ultrafast conditions. The agreement between the two difference absorption spectra indicates that the long-lived photoproduct (likely the *cis* isomer) is formed on a ps timescale and persists to the steady state. The slight discrepancy between the two plots, particularly the peak in the TAS at 365 nm compared to the UV-visible difference spectrum at 370 nm, is attributed to one of two possible effects. Firstly, the blue shift may be due to the involvement of ESA from a populated triplet state. Secondly, whilst the sample volume is replenished from shot to shot, there may be some residual *cis* isomer remaining from previous laser photoexcitation which could contribute to this apparent spectral shift when a change in optical density is calculated.

To garner further insight into the potential photoisomerisation pathway, further structural interrogation of these long-lived photoproducts was achieved through ^1^H spectra taken pre- and post-irradiation with a solar simulator (see Supplementary Figures S10–S13). Looking at the 5.75–7.75 ppm region of the ^1^H spectrum shown in Figure 3, the *cis* isomer is clearly identifiable in the new peaks which have grown following irradiation by approximately the same amount as the original (*trans*) peaks have diminished; the ratio of integrals between *cis* and *trans* peaks is approximately 3:1 following 3 h irradiation in all cases. The chemical shifts (7.15, 6.86, 6.29 and 4.10 compared to 7.47, 6.79, 5.96 and 3.46 ppm) for the new peaks generated are characteristic of the *cis* isomer. The J-values of the *cis* and *trans* isomers are too alike to distinguish in the spectrum. It should be noted that in d_3_-acetonitrile, the three acidic protons on SphA are invisible in the spectra due to exchange with the solvent. No substantial peaks other than those predicted for the *cis* isomer were found in the post-irradiation ^1^H NMR, suggesting that the photoisomerisation yield following excitation by the solar simulator is very high compared to competing photochemical pathways.

The photostability of SphA under a solar spectrum (see Supplementary Figures S14–S16) is comparable to that of similar cinnamate derivatives [29,36]. Based on the NMR data collected for SphA in d_3_-acetonitrile and information from the Δ*t* = 1.0–1.8 ns TAS, we conclude that the observed depletion of absorption at ~290 nm in the UV-visible spectrum is a result of photoisomerisation to a product generated < 1.0 ns after photoexcitation. The resulting *cis* isomer photoproduct has a diminished absorbance profile compared to its *trans* counterpart.

Looking at the TAS qualitatively, the fact that the sequential global fitting analysis yielded three lifetimes (τ_1_, τ_2_ and τ_3_) of approximately the same magnitude in each case indicates that similar photophysical processes are occurring during the relaxation of SphA from its 1^1^ππ* state in each solvent system. It should be noted that the extracted time constants include a convolution with an instrument response function characteristic for each system studied.

Previous solution-phase studies have proposed that following photoexcitation to the 1^1^ππ* state, cinnamates and related compounds typically undergo a rapid geometry change out of the Franck–Condon region, then photoisomerise via an S_1_/S_0_ CI [37]. This initial rapid geometry change, characterised typically by the first lifetime τ_1_, generally appears as a slight spectral change in the early TAS. In this study, however, any fast processes occurring on the <200 fs timescale (a lifetime similar to our instrument response) cannot be deconvolved into distinct steps. Our extracted τ_1_, therefore, probably consists of a convolution of artefacts from the solvent response, geometry relaxation/solvent rearrangement and propagation (in part) through a CI to the S_0_ electronic state [38]. The EADS corresponding to the lifetimes extracted demonstrate a rapid relaxation from the S_1_ state. In acetonitrile, SE centred at 450 nm can be clearly seen beyond 1 ps following photoexcitation, indicating that excited state relaxation dynamics are very fast, but that τ_2_ contains some component of trapped population in the excited state. τ_2_ (~1 ps in all cases) is assigned to the final photodynamic process occurring during the relaxation of SphA: vibrational cooling down the S_0_ surface from the 1^1^ππ*/S_0_ CI. τ_2_ is approximately commensurate with lifetimes attributed to vibrational cooling in past studies [28,37]. It should be noted that, from visual inspection, the global analysis afforded by Glotaran is not capable of totally capturing the vibrational cooling in any solvent system; the ESA blue shifts from ~1 to ~5 ps, but this is not reflected in EADS.

The appearance of the *cis* isomer photoproduct absorption profile at ~350 nm in each system agrees with the photoisomerisation-dominated NRD pathway proposed. After the appearance of this feature (at ~5 ps), the signal gradually narrows and blue shifts, as previously mentioned; this would be expected if the S_0_ were initially populated in a vibrationally hot state which subsequently cools by energy transfer to the immediate solvent environment to form the *cis* isomer [38].

**Figure 5:**
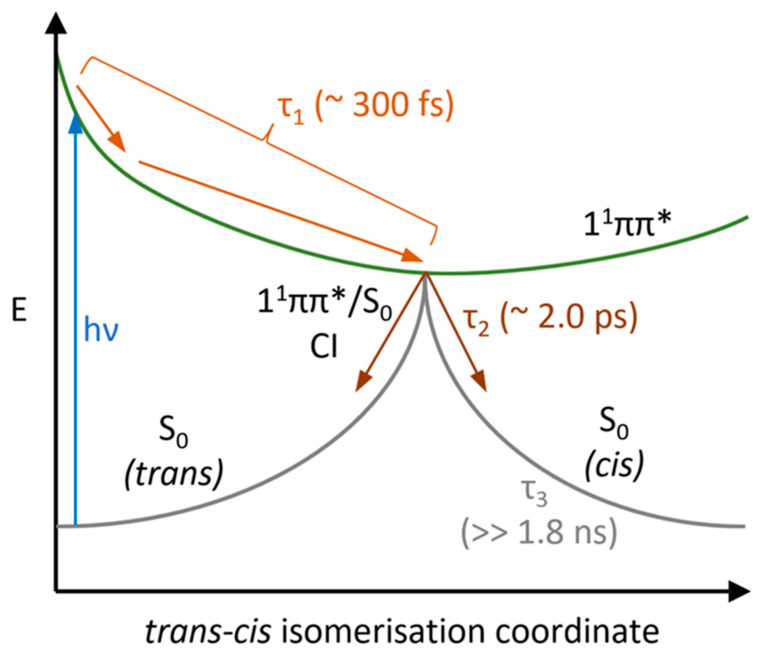
Schematic showing the proposed relaxation mechanism following photoexcitation to the 1^1^ππ* state, with approximate lifetimes for each process identified from TEAS. Although the first two processes occurring (light orange) are separate and theoretically spectrally resolvable, they are convoluted in the first lifetime τ_1_ in our TAS experiment.

As previously mentioned, τ_3_ represents the long-lived vibrationally cool *cis* isomer photoproduct, whose lifetime extends well beyond our experimental Δ*t* window of 1.8 ns. A schematic summary of the processes proposed to occur following photoexcitation of SphA to 1^1^ππ* is shown in Figure 5.

Photophysical relaxation mechanisms hypothesised in previous studies of cinnamates, including ring deformation to reach a CI, are not apparent, but may provide an alternative NRD method for SphA [34,39]. It is not clear whether any photoisomerisation of SphA would result from propagation through this different CI. Surface-hopping and non-adiabatic dynamics simulations are required to generate a more complete image of the potential relaxation mechanisms available to SphA [39]. The observed pre- and post-irradiation ratios of *trans*:*cis* isomers of SphA do, however, demonstrate that photo-induced isomerisation is occurring to an appreciable extent, and so this should lend credence to some understanding of the excited state dynamics of the molecule.

The solvent effects on the relaxation of SphA are not particularly noteworthy: the lifetimes corresponding to the relaxation processes of SphA in acetonitrile are longer than those in ethanol and dioxane. There is no immediate intuitive reason why the photodynamics of SphA are comparatively retarded in the intermediate polarity solvent. This apparent lack of solvent dependence on dynamics is a potential advantage if SphA were used in sunscreens; manufacturers can be confident that SphA’s dynamics are not affected by the solvent environment used in a formulation. This finding forms a segue for future studies into the photodynamics of SphA in water—the solvents used in these studies were selected as models for SphA in non-aqueous sunscreen formulations. The photophysics of SphA in more perturbing solvents where intermolecular interactions may be stronger (i.e., water) may differ from those identified supra. For example, stronger interactions may impact large-amplitude nuclear rearrangement (such as an isomerisation) which could noticeably impact dynamics.

It is apparent that in each solvent case, the NRD dynamics of SphA are fast compared to other UV filters used in sunscreen blends (e.g., ethyl ferulate, avobenzone). Likewise, the excited state dynamics of another plant sunscreen molecule, sinapoyl malate, are slow relative to SphA [40]. It is possible that these accelerated relaxation dynamics are due in part to the steric bulk provided by the ethanoic acid moiety on the alkene leading to diminished coplanarity between the double bond and the phenol group. The energetic barrier in the S_1_ leading to the S_1_/S_0_ CI (and planar symmetry breaking) is, therefore, more surmountable (and repulsive), rendering overall excited state dynamics faster [41]. Based on the observed ultrafast photodynamics and absence of generated deleterious photoproducts, SphA warrants further study in terms of both explaining its dynamics (via ab initio computational studies in an explicit solvent model, rather than in an implicit one (see Supplementary Figures S1–S3)) and investigating its viability for use in sunscreen formulations.

## 4. Materials and Methods

### 4.1. Transient Electronic Absorption Spectroscopy (TEAS)

The experimental setup for fs-TEAS has been described in detail elsewhere and will be summarised herein [35]. Femtosecond laser pulses (3 W, 1 kHz repetition rate) with a central wavelength of 800 nm were generated by a Ti:sapphire regenerative amplifier (Spitfire XP, Spectra-Physics, Milpitas, CA, USA), seeded by a Ti:sapphire oscillator (Tsunami, Spectra-Physics). This train of femtosecond pulses was split into three equivalent 1 W beams, two of which are involved in the TEAS experiment presented herein. One 1 W beam was used to generate ‘*pump*’ pulses, of a desired central wavelength, using an optical parametric amplifier (OPA) (TOPAS-C, Spectra-Physics). The second 1 W beam was further divided into two beams of 0.95 W and 0.05 W, respectively. The 0.05 W beam was focussed into a translating CaF_2_ window to produce a white light supercontinuum (‘*probe*’ pulse, ~315–740 nm). Pump-probe time delay (Δ*t*) was varied by changing the delay of the probe pulse using a gold retroreflector. After passing through the sample, the probe pulse is collimated, goes through an 800 nm filter, and is focussed into a fibre-coupled spectrometer (AvaSpec-ULS1650F, Avantes, Apeldoorn, The Netherlands) by a CaF_2_ lens, from which changes in optical density were determined. The sample (1 mM in each solvent) was circulated in a flow-through cell (Demountable Liquid Cell, Harrick Scientific Products Inc., Pleasantville, NY, USA) consisting of two CaF_2_ windows (0.5 and 1 mm, respectively) spaced 100 μm apart. The irradiated sample was continually replenished at 75 mL/min from a 25 mL reservoir using a peristaltic pump (Masterflex, Gelsenkirchen, Germany). TEAS scans at each time delay were repeated a minimum of 5 times in each solvent system to achieve a reasonable signal-to-noise ratio for each system.

### 4.2. Steady-State Spectroscopy

Steady-state absorption spectra were obtained to assess the photostability of SphA. UV-visible spectra were recorded (Cary 60 UV/Vis spectrophotometer, Agilent, Santa Clara, CA, USA) after irradiation of the solution (~0.03 mM) with a solar simulator (LCS-100, Spectra-Physics).

^1^H NMR (300 MHz, d_3_-acetonitrile) spectra were taken pre- and post-irradiation of SphA to identify the photoproducts generated. SphA was dissolved in deuterated acetonitrile (~20 mM) as the solvent used in the TEAS experiments that was most readily available in the deuterated form. The sample was irradiated with a solar simulator (LCS-100, Spectra-Physics) for 3 h. These NMR spectra were collected twice to ensure *trans*:*cis* isomer ratios were consistent.

### 4.3. Computational Calculations

SphA’s structure was optimised and characterised using harmonic vibrational frequency calculations employing the B3LYP functional in combination with the Dunning basis set cc-pVTZ [42,43,44,45]. Based on the optimised structure, TDDFT calculations were made at the same level of theory and using the polarisable continuum model (PCM) to account for solvent effects of ethanol, acetonitrile, and dioxane, respectively. All calculations were performed with the Gaussian 16 software package [46,47,48,49,50].

## 5. Conclusions

SphA has been studied using TEAS in conjunction with steady-state spectroscopic methods to determine its excited state dynamics in solution. It was determined that excitation to the 1^1^ππ* state drives a *trans*-*cis* photoisomerisation on a ps timescale, mediated by a 1^1^ππ*/S_0_ CI. The resulting photoproduct *cis* isomer is generated in a vibrationally hot state before dissipating its excess energy to the surrounding solvent environment. It was also ascertained that SphA’s fast relaxation rate is largely unaffected by the solvent system. Based on SphA’s absorption profile and photodynamics, we suggest the molecule for use as support to sunscreen formulations containing avobenzone, to combat the generation of deleterious free radicals demonstrated as arising from avobenzone’s exposure to UV-B, as well as providing photoprotection in the UV-B region. As the toxicology of commercial UV filters continues to be unravelled, it is possible that natural alternatives, such as SphA, may supplant other existing sunscreen ingredients (e.g., octocrylene, currently used in formulations containing avobenzone) [51,52,53]. Future work such as ab initio computational studies may provide an understanding of the fast relaxation rates exhibited by SphA. The first look at the ultrafast properties of this molecule from moss has been surprising: for a plant that does not typically grow in direct sunlight, its energy dissipation scheme surpasses that of existing natural plant filters which are thought to absorb UVR in abundance [54]. We believe this is grounds for further research into other moss-based chromophores, to expand the catalogue of nature-inspired compounds capable of supporting UV filters in the continuing search for efficient, inexpensive, non-toxic sunscreen agents.

## Figures and Tables

**Table 1:** Time constants extracted from the sequential global fitting analysis of the collected TAS.

Lifetime	Ethanol	Acetonitrile	Dioxane
τ_1_ (fs)	280 ± 50	330 ± 60	236 ± 50
τ_2_ (ps)	1.31 ± 0.05	3.40 ± 0.06	1.37 ± 0.05
τ_3_ (ns)	>>1.8	>>1.8	>>1.8

## Data Availability

The data presented in this study are available on request from the author (M.H.).

## Supplementary Materials

The following supporting information can be downloaded at: https://www.mdpi.com/article/10.3390/molecules28166153/s1, Figures S1–S3: UV-visible spectra of SphA in ethanol, acetonitrile, dioxane and water, including calculated vertical excitation energies; Figures S4–S6: Residuals for Glotaran fit to TAS data; Figures S7–S9: Instrument response functions (solvent-only TAS); Figures S10–S13: ^1^H NMR and ^1^H-^1^H COSY NMR of SphA in d_3_-acetonitrile pre- and post-irradiation; Figures S14–S16: UV-visible spectra of SphA demonstrating photostability under a solar spectrum.

## Sample Availability

Samples of SphA are available from the authors.
