# Supplementary material for: Characteristic Photoprotective Molecules from the Sphagnum World: A Solution-Phase Ultrafast Study of Sphagnic Acid
Source: Molecules. 2023 Aug 21;28(16):6153. doi: 10.3390/molecules28166153 (PMC10458426; doi:10.3390/molecules28166153)
Supplement: Supplementary file 1 [file molecules-28-06153-s001.zip › molecules-2533164-supplementary.pdf]

Electronic Supplementary Information for:

Characteristic Photoprotective Molecules from the Sphagnum World: A Solution-Phase Ultrafast Study of Sphagnic Acid

Michael Hymas [1], Irene Casademont-Reig [1,2], Stéphane Poigny [3,*] and Vasilios G. Stavros [1,4,*]

1 Department of Chemistry, University of Warwick, Coventry CV4 7AL, UK; michael.hymas@warwick.ac.uk (M.H.); irene.casademont.reig@vub.be (I.C.-R.)
2 Department of General Chemistry (ALGC), Vrije Universiteit Brussel (VUB), Pleinlaan 2, 1050 Brussels, Belgium
3 Mibelle Group Biochemistry, Mibelle AG, Bolimattstrasse 1, CH-5033 Buchs, Switzerland
4 School of Chemistry, University of Birmingham, Edgbaston, Birmingham B15 2TT, UK
* Correspondence: stephane.poigny@mibellegroup.com (S.P.); v.stavros@bham.ac.uk (V.G.S.)

A. UV-vis spectra of sphagnic acid

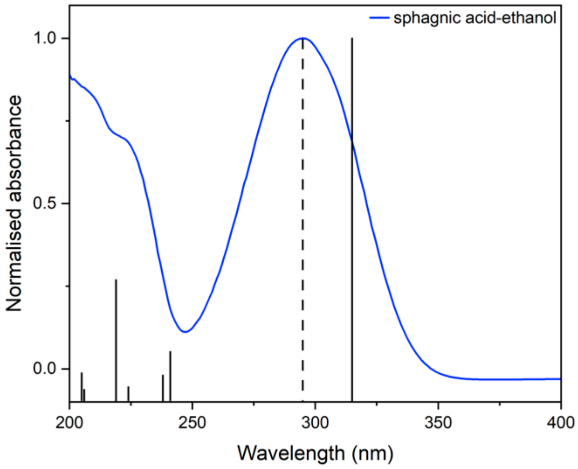

Figure S1: UV-vis spectrum of sphagnic acid in ethanol (obtained using a Cary 60 UV-vis spectrophotometer) (blue); dashed line shows pump wavelength selected for TEAS (295 nm); calculated vertical excitations for sphagnic acid (solid black), computed using B3LYP functional in combination with cc-PVTZ basis set including implicit solvation by ethanol with the PCM model.

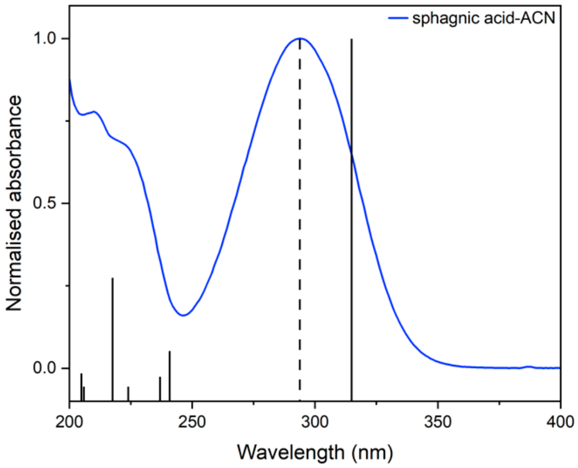

Figure S2: UV-vis spectrum of sphagnic acid in acetonitrile (obtained using a Cary 60 UV-vis spectrophotometer) (blue); dashed line shows pump wavelength selected for TEAS (294 nm); calculated vertical excitations for sphagnic acid (solid black), computed using B3LYP functional in combination with cc-PVTZ basis set including implicit solvation by acetonitrile with the PCM model.

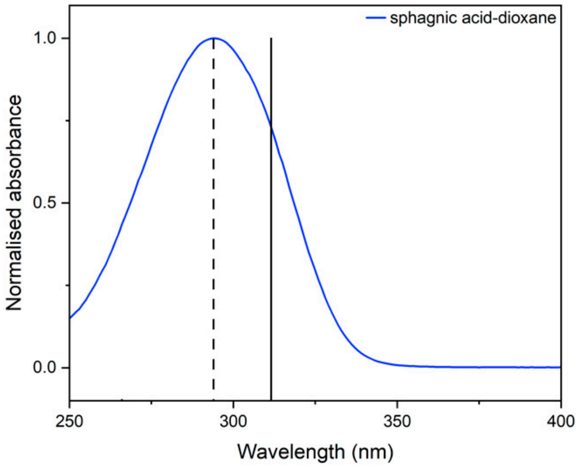

Figure S3: UV-vis spectrum of sphagnic acid in dioxane (obtained using a Cary 60 UV-vis spectrophotometer) (blue); dashed line shows pump wavelength selected for TEAS (294 nm); calculated vertical excitations for sphagnic acid (solid black), computed using B3LYP functional in combination with cc-PVTZ basis set including implicit solvation by dioxane with the PCM model.

B. Residuals from fitting

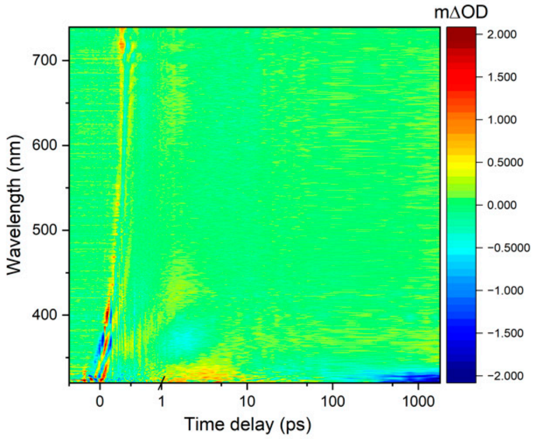

Figure S4: Residuals between Glotaran fits and non-chirp corrected experimental data for sphagnic acid in ethanol

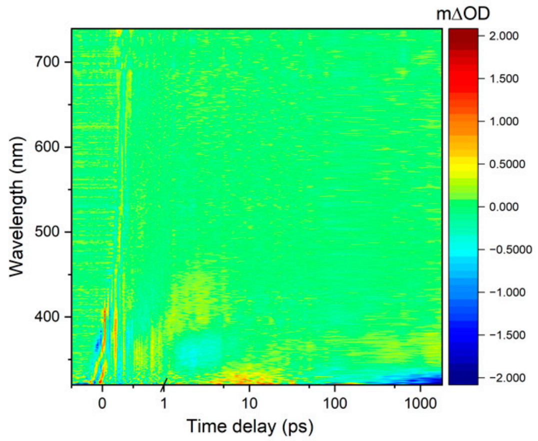

Figure S5: Residuals between Glotaran fits and non-chirp corrected experimental data for sphagnic acid in acetonitrile

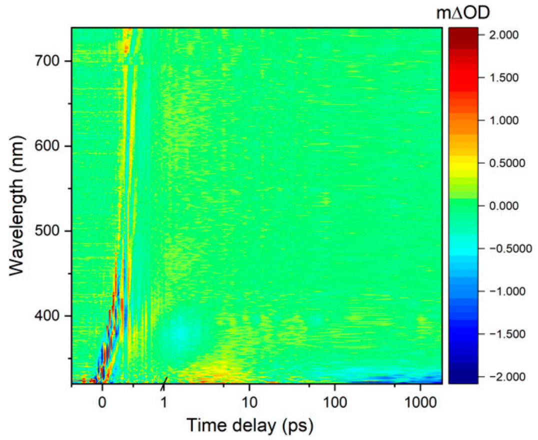

Figure S6: Residuals between Glotaran fits and non-chirp corrected experimental data for sphagnic acid in dioxane

C. Transient absorption spectra of solvent alone

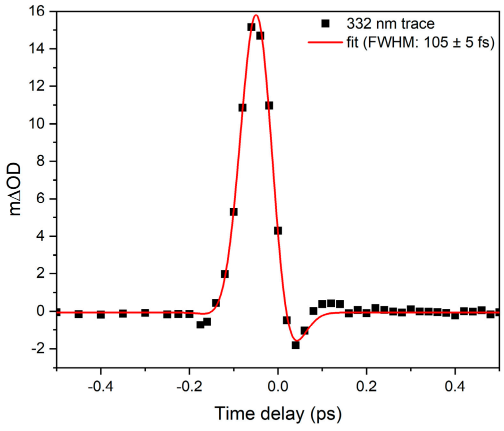

Figure S7: TAS trace at 332 nm of ethanol pumped at 295 nm and fitted with modified Gaussian to gauge instrument response

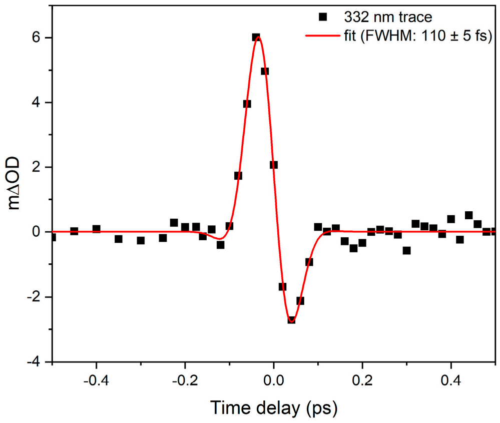

Figure S8: TAS trace at 332 nm of acetonitrile pumped at 294 nm with modified Gaussian fitting to gauge instrument response

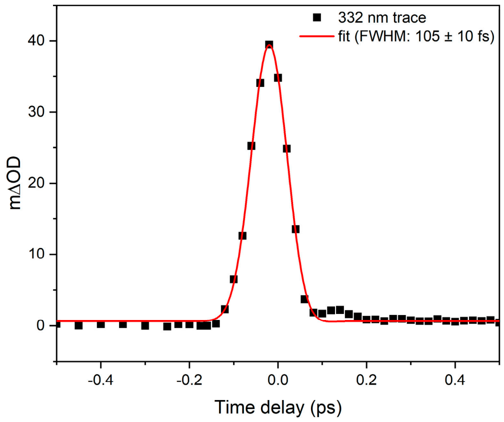

Figure S9: TAS trace at 332 nm of dioxane pumped at 294 nm with modified Gaussian fitting to gauge solvent response

D. NMR

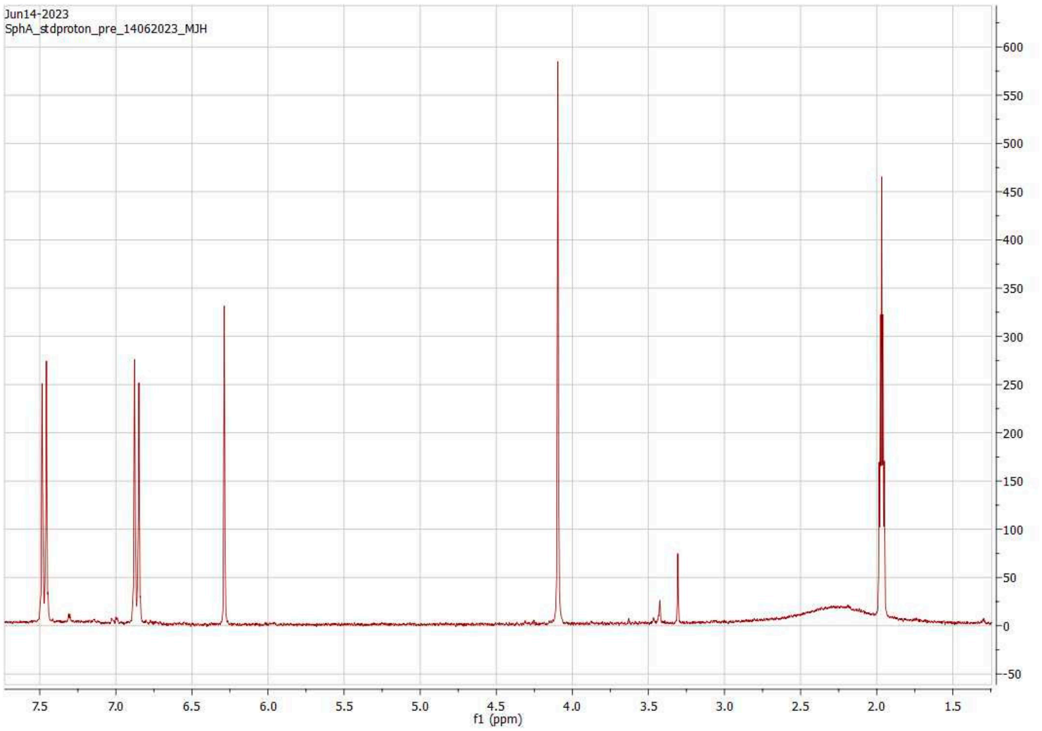

Figure S10: Standard ^{1}H NMR of sphagnic acid in d_3-acetonitrile pre-irradiation with solar simulator

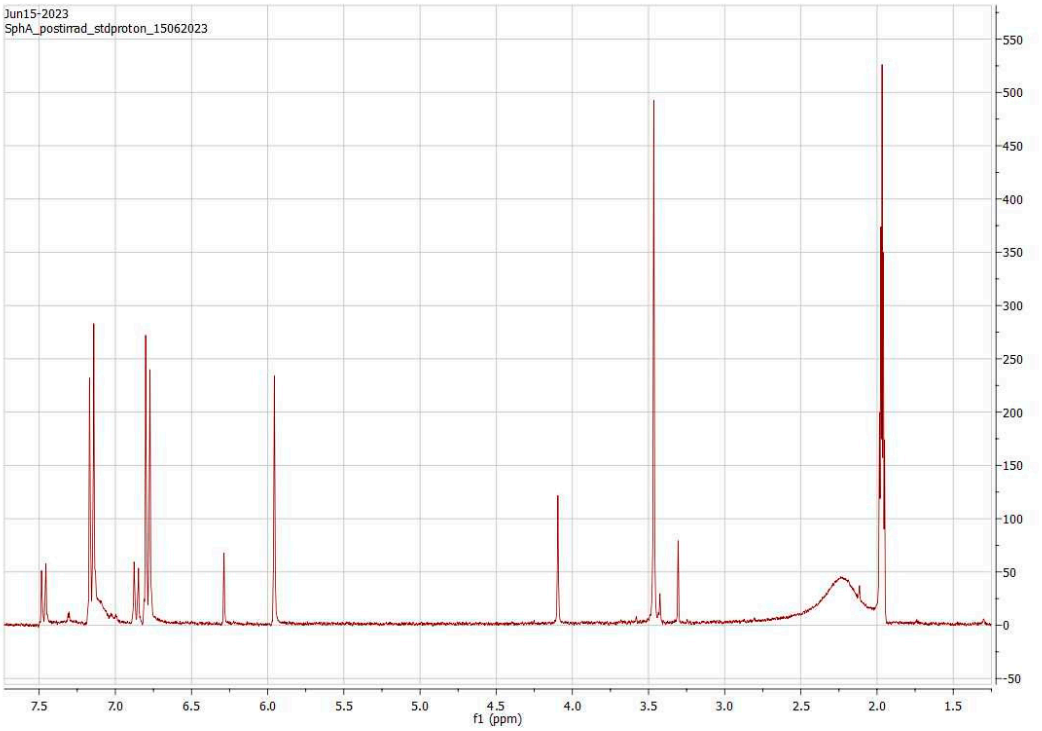

Figure S11: Standard ^{1}H NMR of sphagnic acid in d_3-acetonitrile post-irradiation with solar simulator

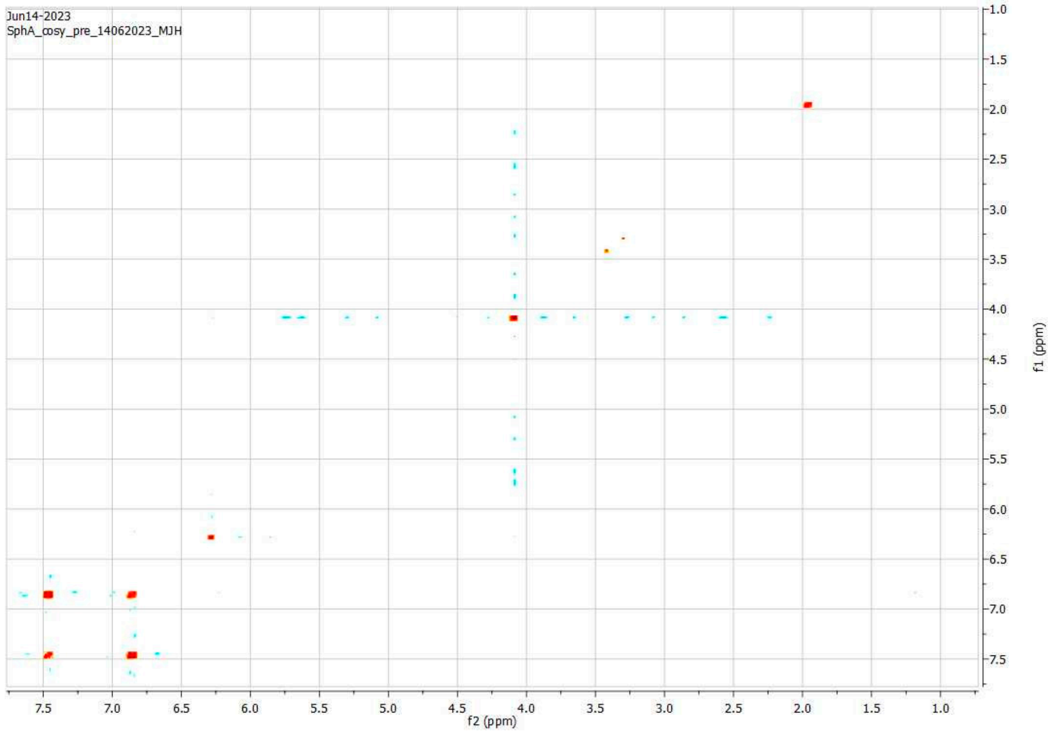

Figure S12: ^{1}H-^{1}H COSY NMR of sphagnic acid in d_3-acetonitrile pre-irradiation with solar simulator

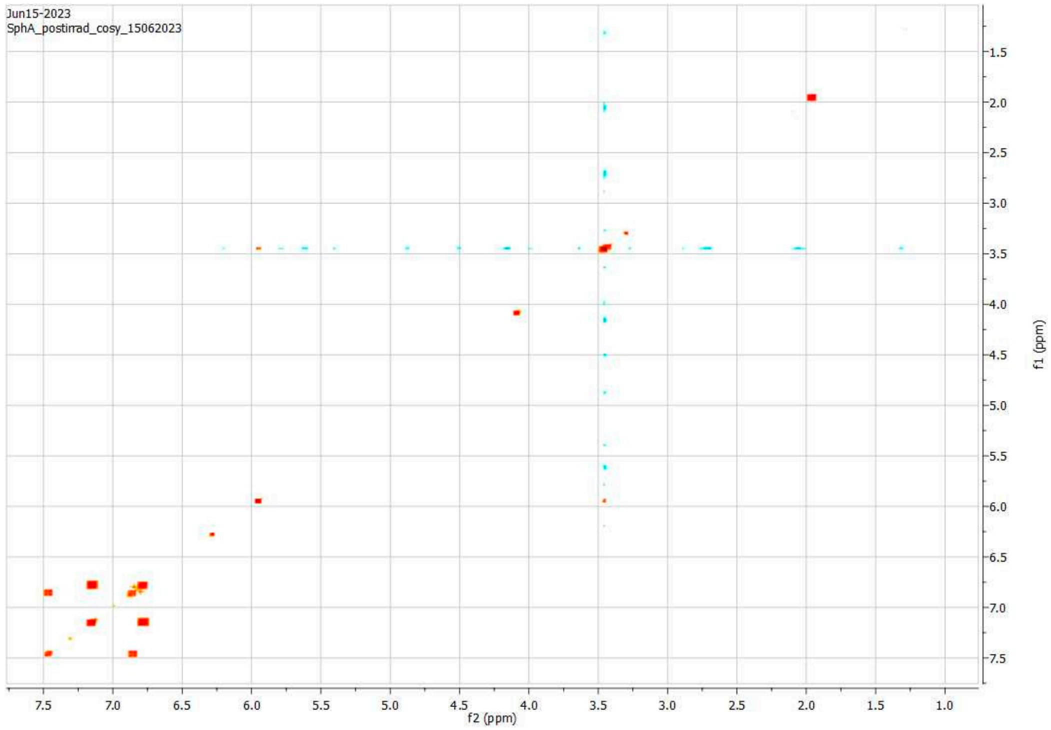

Figure S13: ^{1}H-^{1}H COSY NMR of sphagnic acid in d_3-acetonitrile post-irradiation with solar simulator

E. Stability/photostability of sphagnic acid in solution

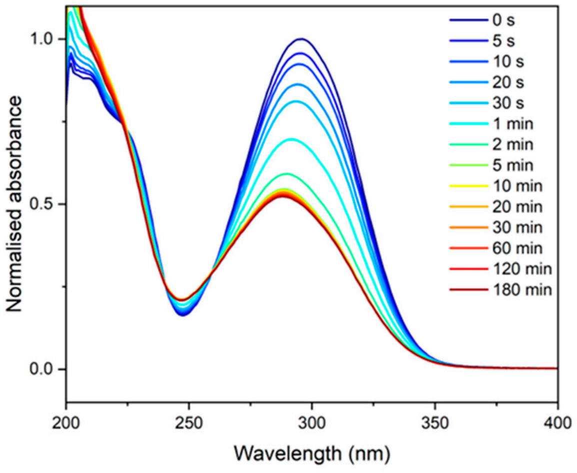

Figure S14: Normalised UV-vis spectra of sphagnic acid in ethanol taken after irradiation at various time intervals using a solar simulator

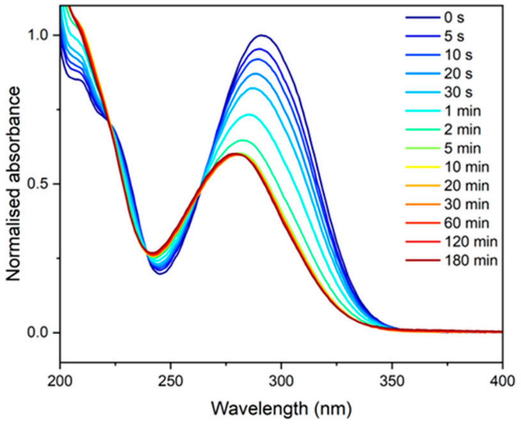

Figure S15: Normalised UV-vis spectra of sphagnic acid in acetonitrile taken after irradiation at various time intervals using a solar simulator

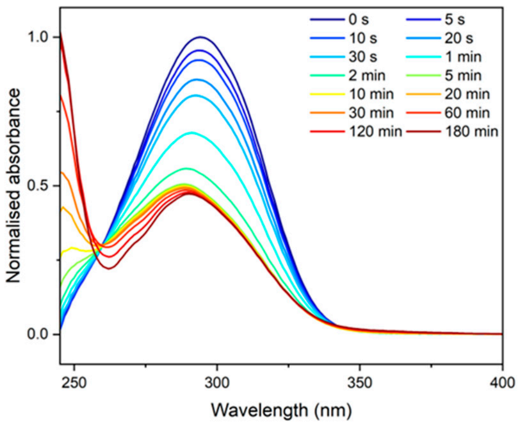

Figure S16: Normalised UV-vis spectra of sphagnic acid in dioxane taken after irradiation at various time intervals using a solar simulator
